# Hernia among Patients Admitted to the Department of Surgery of a Tertiary Care Centre

**DOI:** 10.31729/jnma.8361

**Published:** 2023-12-31

**Authors:** Raj Nandan Yadav, Jemesh Singh Maharjan, Jasmine Bajracharya, Giridhar B.N. Pradhan, Sunil Shrestha

**Affiliations:** 1 Department of Medical Intensive Care Unit, Civil Service Hospital, Minbhawan, Kathmandu, Nepal; 2 Department of General Surgery, Nepal Medical College and Teaching Hospital, Jorpati, Kathmandu, Nepal; 3 Department of Paediatrics Surgery, Nepal Medical College and Teaching Hospital, Jorpati, Kathmandu, Nepal

**Keywords:** *hernia*, *inguinal hernia*, *prevalence*, *surgery*, *umbilical hernia*

## Abstract

**Introduction:**

Hernia is one of the most common surgical conditions causing disability and requiring hospital admission and surgery. The aim of this study was to find out the prevalence of hernia among patients admitted to the Department of Surgery of a tertiary care centre.

**Methods:**

A descriptive cross-sectional study was conducted among patients admitted to the Department of Surgery between 14 April 2021 and 13 April 2023 and were collected from 1 July 2023 to 31 July 2023 from the hospital records. Ethical approval was obtained from the Institutional Review Committee. The patient admitted to the Department of Surgery was included and those with incomplete data were excluded. Convenience sampling was used. The point estimate was calculated at a 95% Confidence Interval.

**Results:**

Out of 2057 patients, the prevalence of hernia was 247 (12.01%) (10.61-13.41, 95% Confidence Interval). A total of 31 (12.55%) hernias were irreducible and 15 (6.07%) were operated in the emergency setting. The most common type of hernia was inguinal hernia found in 169 (68.42%) and hypertension was the most common comorbidities found in 48 (19.43%).

**Conclusions:**

The prevalence of hernia was similar to other studies done in similar settings. Hernia accounts for a major surgical burden in our setting. So, early diagnosis and treatment could reduce the morbidity and mortality related to it.

## INTRODUCTION

A hernia is the bulging of part of the contents of the abdominal cavity through a weakness in the abdominal wall.^1^ Globally, the overall prevalence of abdominal wall hernia is 1.7%, with 4% occurring beyond the age of 45 and annually more than 20 million hernias are operated worldwide.^2^ More than 750,000 abdominal wall hernias in the United States and over 125,000 hernias in the United Kingdom are operated on each year, accounting for 15-18% of all surgical procedures.^3^

In Nepal, the estimated number of people with groin masses is 310,000 and nearly 66,000 males with soft/reducible groin masses are in need of evaluation.^4^ So, the burden of hernia should be evaluated to enhance our knowledge, improve the quality of life of affected patients and reduce years of healthy life lost to disability (YLD).

This study aimed to find out the prevalence of hernia among patients admitted to the Department of Surgery of a tertiary care centre.

## METHODS

This descriptive cross-sectional study was conducted among the patients admitted to the Department of Surgery of the Nepal Medical College and Teaching Hospital, Jorpati, Kathmandu, Nepal. Ethical approval was obtained from the Institutional Review Committee (Reference number: 67/2079/080). Data from 14 April 2021 to 13 April 2023 were collected between 1 July 2023 to 31 July 2023 from the hospital records. During the aforementioned study period, patients who were admitted to the Department of Surgery with complete data were included, and those with missing data were excluded. A convenience sampling method was used. The sample size was calculated with the following formula:


$$\begin{array}{ll} \text{n} & ={\text{Z}}^{2}\times \frac{\text{p}\times \text{q}}{{\text{e}}^{\text{2}}}\\ & =1.96^{2}\times \frac{0.50\times 0.50}{0.05^{2}}\\ & =385 \end{array}$$

Where,

n = minimum required sample size
Z = 1.96 at a 95% Confidence Interval (CI)
p = prevalence taken as 50% for maximum sample size calculation
q = 1-p
e = margin of error, 5%

The computed minimum required sample size was 385. The sample size was quadrupled and the calculated sample size was 1560. However, 2057 patients were included in the study.

Age, sex, types of hernia, reducibility, side of hernia, comorbidities, and outcome of hernia were all documented in a preformed proforma for patients with hernias. Data were entered in Microsoft Excel 2016 and analysed using IBM SPSS Statistics 17.0. The point estimate was calculated at a 95% CI was calculated.

## RESULTS

**Figure 1:**
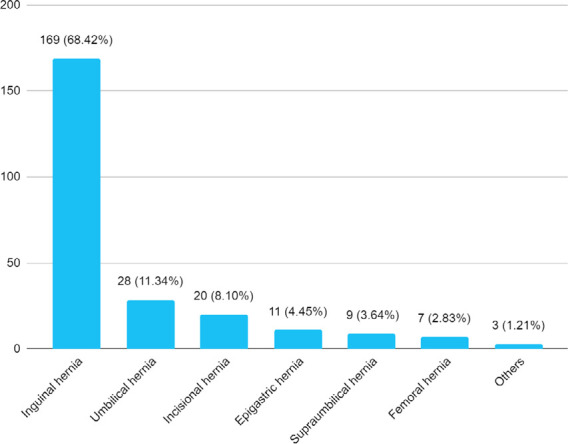
Types of hernia (n= 247).

Out of 2057 patients, the hernia was seen in 247 (12.01%) (10.61-13.41, 95% CI). The mean age of the hernia patients was 42.76±21.85 years. Among them, 216 (87.45%) were reducible and 31 (12.55%) were irreducible. Elective surgery was done in 228 (92.31%) cases, 15 (6.07%) of cases were operated in an emergency setting and 4 (1.62%) hernia cases were managed either conservatively or referred to another centre. The most common hernia was an inguinal hernia found in 169 (68.42%) followed by an umbilical hernia 28 (11.33%) and an incisional hernia 20 (8.09%) (Figure 1).

There were 187 (75.71%) males and 60 (24.29%) females with male:female ratio of overall hernia patients 3.12:1, inguinal hernia 11.07:1, umbilical hernia 1.8:1, incisional hernia 1:5.67, epigastric hernia 1.75:1, supraumbilical hernia 1.25:1. Femoral hernia was seen only in females (Table 1).

**Table 1:** Sex and age-wise distribution of patients with hernia (n= 247).

Variable	Inguinal hernia n (%)	Umbilical hernia n (%)	Incisional hernia n (%)	Epigastric Suprauumbilical hernia n (%)	Femoral hernia n (%)	Others n (%)	Total
**Sex**								
Male	155 (91.72)	18 (64.29)	3 (15)	7 (63.63)	4 (44.44)	-	-	187 (75.71)
Female	14 (8.28)	10 (35.71)	17 (85)	4 (36.37)	5 (55.56)	7 (100)	3 (100)	60 (24.29)
**Age group (years)**								
0-17	37 (21.89)	1 (3.57)	-	-	-	-	-	38 (15.4)
18-39	36 (21.30)	10 (35.71)	1 (5)	2 (18.18)	7 (77.78)	-	1 (33.3)	57 (23.1)
40-59	47 (27.81)	13 (46.43)	12 (60)	7 (63.64)	-	5 (71.43)	2 (66.7)	86 (34.8)
60-79	46 (27.22)	4 (14.29)	7 (35)	2 (18.18)	2 (22.22)	2 (28.57)	-	63 (25.5)
>80	3 (1.78)	-	-	-	-	-	-	3 (1.2)
Total	169 (68.42)	28 (11.34)	20 (8.09)	11 (4.45)	9 (3.65)	7 (2.83)	3 (1.22)	247 (100)

Out of the participants with inguinal hernia, 102 (60.36%) were on the right side (Table 2).

**Table 2:** Distribution of inguinal hernia and femoral hernia (n= 247).

Side	Inguinal hernia n (%)	Femoral hernia n (%)
Right	102 (60.36)	6 (85.71)
Left	49 (28.99)	1 (14.29)
Bilateral	18 (10.65)	-

Out of 169 inguinal hernias, 86 (50.89%) were indirect, 29 (17.16%) were direct, 18 (10.66%) were pantaloon and 36 (21.29%) were congenital inguinal hernia. The most common comorbidity was hypertension found in 48 (19.43%) patients (Table 3).

**Table 3:** Comorbidities among patients with hernia (n= 247).

Comorbidities	n (%)
Hypertension	48 (19.43)
Type 2 DM	16 (6.48)
COPD	12 (4.86)
Hydrocele	9 (3.64)
Hypothyroidism	9 (3.64)
Prostatomegaly	7 (2.83)
Others	49 (19.84)

## DISCUSSION

The prevalence of hernia in our study was 12.01%. The prevalence in a similar descriptive research conducted in a tertiary care facility in Northwest Ethiopia was close to our study at 11.7%.^5^ Another study at the National Academy of Medical Sciences in Kathmandu found a greater prevalence of hernias (23.14%) than in our study.^6^ Saudi Arabian people had a hernia prevalence of 11.7%,^7^ whereas Central Russians had a hernia prevalence of 20.9%.^8^ A tertiary care centre in India reported a 22% burden of abdominal hernia among patients who had been admitted.^9^ About 7.7% was the pooled prevalence of inguinal hernia, according to a meta-analysis.^10^

The most common hernia in our study was an inguinal hernia (68.42%) followed by an umbilical hernia (11.34%) and an incisional hernia (8.09%) (Figure 1). The epigastric hernia had the highest prevalence 34% followed by inguinal hernia 29.8% in the study in Northwest Ehiopia^5^ whereas the most common hernias were para-umbilical (33.9%), inguinal (27.3%), and umbilical (20.8%) in Northern Saudi Arabia.^7^ The inguinal hernia was the most common type found in 77.25%, followed by an umbilical hernia in 8.61%.^6^ Umbilical hernias were found in 10.2%, groin hernias in 8.3%, and incisional hernias in 2.4% of the Russian population.^8^

In our study, the hernia was almost 3 times more common in males than females which was in coherence with other studies in which there were 63.4% males and 36.6% females,^9^ 79.2% males and 20.8% female,^11^ 86.54% males and 13.45% females.^6^ Inguinal, umbilical, epigastric hernia, and supraumbilical hernia showed male preponderance whereas incisional hernia was more common in females, the femoral hernia was seen only in females which were in coherence with the findings of another study in which the male: female ratio was 23.95:1 for inguinal hernia, 1.78:1 for umbilical hernia, and 2.77:1 for an epigastric hernia.^6^

The mean age of the hernia patients was 42.76±21.85 years with the distribution of hernia increasing with age, peaking at age 40-59 years, then falling gradually. Our study was in coherence with the study done at the National Academy for Medical Sciences, Kathmandu6 but was in contrast to another study which shows a a bimodal distribution, with peaks around age 5 and after age 70.^12^

In our study, the inguinal hernia was twice as common on the right side than on the left. Another study showed that 54.1% of the inguinal hernia was on the right, 39.7% was on the left, and 6.2% was bilateral.^13^ Similar findings were seen in a study in northern Jordan with the incidence of right-sided, left-sided, and bilateral inguinal hernias as 60%, 35%, and 5% respectively.^14^

Indirect inguinal hernia was three times more common than direct inguinal hernia in our study. In, a three-year audit, indirect inguinal hernias are twice as common as direct hernias.^15^ Hypertension was the most common comorbidities in our study present in 19.43% of hernia patients compared to 3.34% of hypertensive hernia patients in another study.^6^

The study was single-centred and retrospective, so, it cannot be inferred to a larger population. A multicentred and prospective study could be done to generalize the study.

## CONCLUSIONS

The prevalence of hernia was similar to other studies done in similar settings. Necessary healthcare facilities and human resources could be considered for early diagnosis and management of hernia to decrease morbidity and mortality in patients.
